# The challenges for early intervention and its effects on the prognosis of autism spectrum disorder: a systematic review

**DOI:** 10.1590/1980-5764-DN-2023-0034

**Published:** 2024-02-09

**Authors:** Jackson Frederico Pires, Caroline Cajuela Grattão, Regiane Maria Ribeiro Gomes

**Affiliations:** 1 Universidade Nove de Julho, Faculdade de Medicina, Bauru SP, Brazil.; 2 Universidade Estácio de Sá, Petrópolis RJ, Brazil.

**Keywords:** Autism Spectrum Disorder, Autistic Disorder, Early Medical Intervention, Prognosis, Transtorno do Espectro Autista, Transtorno Autístico, Intervenção Médica Precoce, Prognóstico

## Abstract

Autism spectrum disorder (ASD) is expressed with neurobehavioral symptoms of different degrees of intensity. It is estimated that, for every three cases detected, there are two cases that reach adulthood without treatment.

**Objective:**

To establish what challenges are still present in the implementation of early intervention (EI) and its effects on the prognosis of ASD.

**Methods:**

A systematic review using the Preferred Reporting Items for Systematic Reviews and Meta-Analyses (Prisma) methodology was carried out in the PubMed and ScienceDirect databases in January 2023. The search keywords were “autism spectrum disorder”, “early intervention” and “prognosis”.

**Results:**

Sixteen studies were included, two randomized and 14 non-randomized. Knowledge about the signs of ASD, diagnostic and therapeutic methods, age at the start of treatment, and socioeconomic factors were the main challenges encountered in the implementation of the EI.

**Conclusion:**

EI is capable of modifying the prognosis of ASD and challenges in its implementation persist, especially in developing regions with low socioeconomic status.

## RESUMO.

O transtorno do espectro autista (TEA) expressa-se com sintomas neurocomportamentais de diferentes graus de intensidade. Estima-se que, para cada três casos detectados, existam dois casos que atingem a idade adulta sem tratamento.

**Objetivo:**

Estabelecer quais são os desafios ainda presentes na implementação efetiva da intervenção precoce (IP) e quais são os seus efeitos no prognóstico do TEA.

**Métodos:**

Revisão sistemática com a metodologia *Preferred Reporting Items for Systematic Reviews and Meta-Analyses* (Prisma) foi realizada nos bancos de dados PubMed e ScienceDirect em janeiro de 2023. As palavras-chave da pesquisa foram “autism spectrum disorder”, “early intervention” e “prognosis”.

**Resultados:**

Dezesseis estudos foram incluídos, sendo dois randomizados e 14 não randomizados. O conhecimento sobre os sinais do TEA, os métodos diagnósticos e terapêuticos, a idade de início de tratamento e os fatores socioeconômicos foram os principais desafios encontrados na implementação da IP.

**Conclusão:**

A IP é capaz de modificar o prognóstico do TEA e os desafios em sua implementação ainda afetam, principalmente, regiões em desenvolvimento e de baixo *status* socioeconômico.

## INTRODUCTION

Autistic spectrum disorder (ASD) is a multifactorial neurodevelopmental disorder with strong genetic influence^1^ that is expressed in neurobehavioral symptoms of different degrees of intensity, with manifestations of a classic triad that comprises communication, social interaction, and the execution of stereotyped repetitive behaviors^2^.

In the last decade, there has been a considerable increase in ASD diagnoses, with the highest prevalence observed in males^3^ ^,^ ^4^ and, in addition, there was a high probability of these individuals being diagnosed with other developmental disorders and psychiatric disorders^4^ ^,^ ^5^.

Several theories as to the neuropathology of ASD have been raised, so that reaching a consensus is difficult due to the heterogeneity of factors, such as genetic mutations, the clinical condition of each individual, alterations in the brain anatomy observed in post-mortem studies, and the gender of each patient^6^ ^,^ ^7^.

Inheritance of the ASD-related gene is considered common based on the more than one hundred genes and genomic regions associated with ASD. However, when discovering a rare mutation, geneticists considered a functional effect on the protein-coding regions of the genome that are associated with a greater chance of ASD; however, the contribution of this gene to the risk calculation of ASD is around 3%^6^. The cerebellum is the main region of the brain affected in non-motor disorders. Significant changes likely occur following multiple genetic insults in the structure and function of the cerebro-cerebellar closed circuits, resulting in sensory, motor, and cognitive dysfunction^7^ ^,^ ^8^ ^,^ ^9^.

It should be noted that the proper recognition, as well as the determination of the diagnosis of ASD cases, depends on the clinical correlation and the individuality of each patient. However, the heterogeneity of symptoms made the diagnosis more complex and less accurate. Thus, the American Psychiatric Association standardized diagnostic guidelines with the publication of the Diagnostic and Statistical Manual of Mental Disorders (DSM-5), which mainly considers social and cognitive symptoms and, through tests and questionnaires, consolidated the diagnosis of ASD^10^ ^,^ ^11^ ^,^ ^12^.

However, despite this standardization, many patients reach adulthood without a defined diagnosis, or even without any diagnosis at all^13^ ^,^ ^14^. The prevalence is approximately one in every hundred children diagnosed with ASD in the world. Still, it is estimated that the prevalence has increased over time and has varied greatly among sociodemographic groups^15^. and that ASD affects one in 36 children^16^ and one in one hundred adults. However, for every three cases of ASD detected, there are two undiagnosed ones^17^.

Much has been said about early intervention (EI) as a treatment approach that can change the prognosis of ASD, as well as potentially more effective screening, diagnosis, and intervention tools for this disorder. Due to being a complex disorder with variable severity and symptomatology, the prognosis of patients with ASD remains highly subjective and influenced by various factors. However, assessing the prognosis based on the stratification of symptoms such as language and communication, stereotypies, social skills, and cognition can serve as valuable prognostic indicators.

Previous reviews have quantified the prognostic outcomes regarding symptoms of ASD in the context of different types of interventions. Our goal, therefore, is not only to report the key indicators of a favorable prognosis but also to identify the factors that still influence the achievement of such an outcome; in other words, what factors still hinder its occurrence. This review seeks to identify in the current literature the effects of EI in cases of ASD, in order to seek answers to questions that are still relevant, involving the ideal age to obtain a reliable diagnosis, how much the age at diagnosis alters the prognosis of patients with ASD and other factors that may influence EI.

The object of this systematic review is significant at a time when its results may contribute to guiding new scientific research involving EI strategies and changes in prognosis, as well as directing effective public policies that aim to improve care for this population, mainly in vulnerable situations and in developing countries that lack efficient strategies and resources.

## METHODS

This article presents a systematic review based on the analysis of scientific articles published between 2013 to 2023, in PubMed and ScienceDirect, carried out using the Patient or Population, Investigation/Interest, Comparison, Outcome, and Study Design (PICOS) strategy^18^. The following aspects were included in this systematic review: the patients with ASD (population), challenges for the implementation of effective early intervention (research/interest), use of different intervention methods, with or without parental participation, in situations of high and low socioeconomic status (comparison), delay in early intervention, neurodevelopmental delay, and symptom exacerbation (outcome), and randomized, non-randomized and case-control studies (study design). According to the pillars defined for the establishment of PICOS, it was possible to define the central question of this review: What are the still persistent challenges for the implementation of effective early intervention in the population with ASD? This systematic review was performed according to the Preferred Reporting Items for Systematic Reviews and Meta-Analyses (PRISMA) checklist^19^.

### Search strategy and selection criteria

Applying the Boolean operator AND, a search was conducted with the following keywords: “autism spectrum disorder”, “early intervention”, and “prognosis”. The inclusion criteria were:

- original article, reviews or case reports published in the past ten years (January 2013 to January 2023);
- published in English;
- studies on the influence of early intervention on the prognosis of ASD; and
- studies on interferences in early intervention for ASD.

The exclusion criteria were:

- monographs, dissertations, reviews, theses, book chapters and research protocols still under development;
- studies that did not mention the theme “The effects and challenges of early intervention on autism spectrum disorder”;
- articles that were not found in English;
- articles that exclusively reported screening studies and diagnostic tests; and
- articles that reported only risk analysis for ASD.

### Study selection

The selection of studies was performed in three phases (identification, screening and inclusion). In the first phase, two reviewers (CCG and RMRG) performed a search of all the electronic databases adopted in our investigation. The reviewers (CCG and RMRG) independently reviewed the titles and abstracts of all electronic citations from databases related to the study. Articles that objectively did not meet the inclusion criteria were excluded.

In the second phase, an independent reviewer (JFP) analyzed the articles preliminarily selected according to the established inclusion criteria and the references were retrieved. In the first and second phases of this search protocol, disagreements among the three reviewers (JFP, CCG and RMRG) were resolved by consensus.

In the third phase, all previously screened articles had their eligibility confirmed or not after the complete reading of the text. This step was performed independently by two reviewers (JFP and CCG), standardized by a previously defined form with the eligibility criteria. Any disagreements were resolved by consensus. Agreement between raters in the selection of studies was assessed using Cohen’s Kappa coefficient.^20^

### Data extraction

Two reviewers (JFP and CCG) extracted the data using a table where they included the name of the first author, year of publication, kind of study, sample size and characteristics, study objective and method, main results and conclusions. Any disagreement during data extraction was resolved by consensus.

### Study risk of bias assessment

To assess the risk of bias (RoB) of the included studies, the Mixed Methods Appraisal Tool (MMAT, version 2018)^21^ was adapted for the present study and used as the review instrument. The assessment of RoB was conducted using the screening questions from items 2 and 3 of the MMAT, according to the design of the selected study, as follows: (Item 2) Quantitative randomized controlled trials; and (Item 3) Quantitative non-randomized studies.

The responses obtained from the MMAT were classified as “yes”, “no”, or “cannot tell”, and were categorized into five levels of evidence based on the percentage of criteria met (20%, 40%, 60%, 80% and 100%). Studies received 20 percentage points for each “yes” response. In order to create a visual representation of the RoB, the RevMan software (Review Manager, software version 5.4.1, Cochrane Collaboration, Copenhagen, Denmark) was used, with modifications made to align with the MMAT questions. The responses from the MMAT tool (“yes”, “no”, or “cannot tell”) were interpreted as indicating low, high, or unclear risk, respectively, in the RevMan table for assessing the RoB. Higher percentages indicate greater quality, with 100% indicating that all quality criteria were satisfied. The MMAT was administered by the first author (JFP), with the third author (RMRG) examining the first 15% of the included studies. Upon reaching 100% agreement regarding the application of the rating criteria, the primary reviewer (JFP) proceeded to independently apply the tool to the remaining studies.

## RESULTS

**Figure 1.**
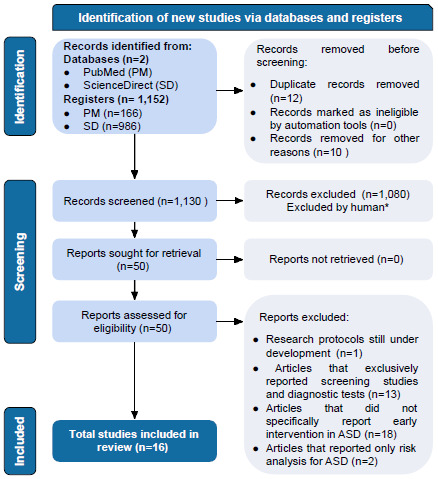
The article selection process according to the Preferred Reporting Items for Systematic Reviews and Meta-Analyses initiative recommendations.

Abbreviations: ASD, Autism spectrum disorder; PM, PubMed; SD, Science Direct.

Based on the Medical Subject Headings (MeSH) terms initially used, this systematic review retrieved 1,152 article records (Figure 1), with 166 and 986 retrieved, respectively, from the PubMed and ScienceDirect databases. In the PRISMA identification stage, 22 articles were excluded because they were duplicates (n=12) or because they were articles that addressed other diseases without including ASD (n=10). Thus, 1,130 articles were registered for the screening phase.

According to the title and/or abstract of the remaining 1,130 articles, 1,080 papers not related to EI and its effects on ASD, articles not published in English or even unpublished studies were excluded, leaving 50 articles.

The complete files of the articles were obtained (n=50). Of these 50 remaining articles, research protocols still under development (n=1) were excluded. Still in this phase of complete reading of the articles, studies that exclusively reported screening exams and diagnostic tests (n=13), but that had no correlation with the application of these tests and changes in prognosis were excluded. Articles that did not specifically report EI in ASD (n=18), that is, which addressed other issues of the disorder such as its relationships with other disorders were also excluded. Articles that only reported risk analysis for ASD (n=2) were excluded too. After selection was carried out independently by each researcher, Cohen’s Kappa coefficient was obtained with an approximate value of 0.66, which can be considered a substantial agreement between the evaluators^22^.

Finally, a total of 16 articles were included in this systematic review study, and the main findings are included in Table 1.

**Table 1.** Detailed representation of studies.

Author	Year	Study design	Sample	Objective	Main results	MMAT score (%)
Clark et al.^23^	2018	Quantitative non-randomized	Experimental (n=48) Comparison (n=37)	Comparison of prognoses according to the initiation of EI for ASD at different ages	Intervention for ASD was most effective in children diagnosed up to 2 years of age.	80
Maksimovic et al.^24^	2023	Quantitative non-randomized	Experimental (n=29)	Comparison of prognoses according to the initiation of EI for ASD at different ages	Autistic symptoms are reduced more in younger children than in older children. EI had better effects in younger children.	100
Lombardo et al.^25^	2021	Quantitative non-randomized	Experimental (n=41)	Comparison of prognoses according to the initiation of EI for ASD at different ages	Shows the importance of early treatment starting ideally before 24 months. Also shows for the first time that blood gene expression characteristics can predict how fast toddlers with ASD respond to early treatment.	60
Vinen et al.^26^	2018	Quantitative non-randomized	Experimental (n=31) Comparison (n=28)	Comparison of prognoses according to the initiation of EI for ASD at different ages	The results of school-aged children with ASD who received EI during their preschool years are promising.	80
Green et al.^27^	2015	Quantitative randomized controlled trials	Intervention (n=28) Control (n=26)	Establishment of prognostic gains from different intervention programs	The specific results suggest that the intervention increased the baby’s attention to parents. Parent-mediated intervention was applied.	100
Frazier et al.^28^	2021	Quantitative non-randomized	Experimental (n=131)	Establishment of prognostic gains from different intervention programs	The EIBI method demonstrated significant changes in the prognosis of ASD, mainly with regard to language. More representative surveys are needed.	60
MacDonald et al.^29^	2014	Quantitative non-randomized	Experimental (n=83) Comparison (n=58)	Establishment of prognostic gains from different intervention programs	Significant gains with EIBI, especially when applied before 24 months	60
Waters et al.^30^	2020	Quantitative non-randomized	Experimental (n=48) Comparison (n=46)	Establishment of prognostic gains from different intervention programs	The study demonstrates that the EIBI method is effective in community settings for children with ASD starting an intervention in different ages throughout early childhood.	100
Howard et al.^31^	2014	Quantitative non-randomized	Experimental (n=29) Comparison (n=32)	Establishment of prognostic gains from different intervention programs	ABA therapy achieved better results than eclectic therapies.	100
Rahman et al.^32^	2016	Quantitative randomized controlled trials	Intervention (n=32) Control (n=33)	Establishment of prognostic gains from different intervention programs	Use of a tailored (parent-mediated) intervention was effective in low- and middle-income countries.	100
Perera et al.^33^	2016	Quantitative non-randomized	Experimental (n=62) Comparison (n=42)	Establishment of prognostic gains from different intervention programs	The home EI results found a statistically significant improvement between pre- and post-intervention in all measured parameters.	80
Kitzerow et al.^34^	2020	Quantitative non-randomized	Case (n=20) Control (n=20)	Establishment of prognostic gains from different intervention programs	The low-intensity early intervention called A-FFIP was effective and brought important prognostic results.	80
Estes et al.^35^	2015	Quantitative non-randomized	Experimental (n=24) Comparison (n=24)	Assessment of whether early intervention results are maintained over time	There is evidence that gains from early intensive intervention are maintained for at least 2 years afterwards.	100
Wei et al.^36^	2022	Quantitative non-randomized *A Cross-sectional analytic study*	Medical Workers (n=269) Educators (n=181) Community (n=188)	Knowledge survey about ASD in people who work or will have early contact with children with ASD	Professionals were able to recognize early signs of ASD but had an inadequate understanding of the disorder.	80
Coelho et al.^37^	2021	Quantitative non-randomized	Experimental (n=55)	Investigation of the development trajectory of two groups of children with ASD in search of predictive factors and adjustments for better intervention strategies	Diagnosis and early intervention are determinants of different prognoses. Parent participation is critical to the success of EI.	80
Jonsdottir et al.^38^	2020	Quantitative non-randomized	Experimental (n=1,586)	Study on screening method and its effects on final prognosis	The M-CHAT-R method was able to detect cases of underdiagnosis at 30 months of age.	60

Abbreviations: MMAT, Mixed Methods Appraisal Tool; ASD, Autism Spectrum Disorder; EI, Early Intervention; EIBI, Early Intensive Behavioral Intervention; ABA, Applied Behavior Analysis.

### Study design and risk of bias in studies

The 16 articles included in this review are quantitative studies, two of them randomized controlled trials and 14 non-randomized studies. All articles underwent rigorous methodological evaluation using the MMAT instrument, in an attempt to quantify possible RoB.

**Figure 2.**
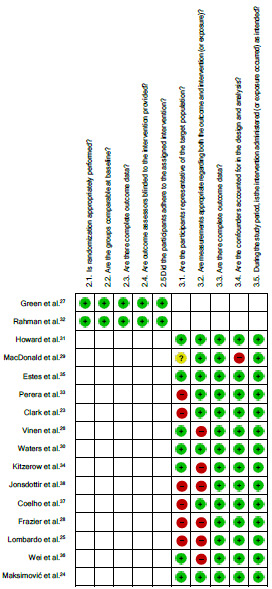
Risk of bias summary.

Figure 2 shows a tab describing the RoB assessment by the RevMan software, with each bias item expressed in all studies included in the analysis. These assessments revealed that four studies were classified as having a moderate RoB, with a score of 60%, and 12 studies were classified as having a low RoB, with a score of 80%, and six studies had achieved 100% on the MMAT scale (Table 1).

### Selected studies and main findings

For organizational reasons, we divided the 16 articles included in this review according to the study objectives of each one of them. In this sense, the main research objectives found were:

1. Studies that compared prognoses according to the initiation of EI for ASD at different ages^23^ ^-^ ^26^;
2. Studies that sought to establish prognostic gains in the face of different intervention programs^27^ ^-^ ^34^;
3. Studies that observed whether there was maintenance of EI results over time^35^;
4. Studies on the knowledge of professionals about the disease^36^;
5. Studies that sought to establish predictive factors for intervention adjustments^37^, and;
6. Studies on screening method and its effects on final prognosis^38^.

The main aspects of each of these studies are better seen in Table 1.

In general, the selected studies agree that EI is essential for the improvement of ASD symptoms and contributes significantly to changing the prognosis of the disorder. Among the studies comparing age at the beginning of the intervention (1), Maksimović et al.^24^ emphasize the importance of specifying the exact age when using the terms “early intervention” and “early development” as a measure to determine exactly when to start the intervention. Clark et al.^23^, reported that children diagnosed early at two years of age had a better prognosis than children diagnosed at three years of age. Lombardo et al.^25^ agree and even suggest that the ideal age to start the intervention is earlier than 24 months, in addition to reporting for the first time the possibility of using gene expression in the blood to detect response to early intervention. All these studies are followed by Vinen et al.^26^_,_ who consider promising results of EI applied to children of preschool age.

In a search to determine the gains from the different modalities of EI (2), Howard et al.^31^ reported greater gains with intensive behavior analytic intervention when compared to other eclectic therapies. Similarly, Frazier et al.^28^, MacDonald et al.^29^, and Waters et al.^30^, evaluated the effectiveness of and compared the intensive intervention methods (EIBI) with other methods, reporting significant gains, mainly related to language^28^ ^,^ ^29^ and reduction of stereotyped behaviors^29^. In addition, it was concluded that early age is a predictor of greater gains^28^ ^,^ ^29^; however, without defining the exact age, preschool age was chosen as the ideal age group for the application of the EI.

Considering the need to adapt the methods to realities where human and socioeconomic resources are lower, Kitzerow et al.^34^ demonstrated the effectiveness of a low-intensity intervention method (A-FFIP), with important cognitive effects. With the same objective, other authors such as Green et al.^27^, Rahman et al.^32^, and Perera et al.^33^ described the importance of parent-mediated methods in home settings, followed by Coelho et al.^37^, who demonstrated the importance of parental perception as predictors for ASD (5).

Estes et al.^35^, in a pioneering study, reported the sustained effects of EI for at least two years (3). Wei et al.^36^, in turn, demonstrated failures in early detection resulting from lack of knowledge of warning signs by professionals who work with the population at risk for ASD (4).

The study by Jonsdottir et al.^38^ is the only one included in this review that involves a screening method, as it specifically addresses correlated changes in prognosis for ASD (6).

## DISCUSSION

### Current overview of diagnosis and intervention

Every year, numerous studies and protocols seek to understand and diagnose individuals with ASD at an early stage, but, despite all the work, many cases are still neglected, impacting the life and development of each patient who stopped receiving his diagnosis^17^. The main objective of rapid ASD detection is EI and the consequent prevention of disabilities related to this disorder.

The American Academy of Neurology, in order to promote a more efficient diagnosis, developed a guide based on two levels to help health professionals in early diagnosis. Thus, level 1 consists of observing the child in primary health care, for example, in child care consultations, to select those who are at high risk of ASD, in addition to monitoring, at each consultation, whether the child’s developmental milestones are being met. In the scope of level 2, after screening is carried out, the patient is referred to specialized professionals for the application of tests and confirmation of the diagnosis^36^. Each of these levels has its assessment tools, to be elucidated in this review.

In fact, medical professionals are able to identify the main symptoms of this disorder and effectively and safely establish a diagnosis at around two years of age and, with the support of standard screening tools, even before 24 months. However, what is commonly observed in practice is that this process is often delayed, so the current average age for diagnosis is around four to five years old^39^. Considering that early diagnosis is what leads to EI, it can be said that recognition of the disorder is relevant in the prognosis of ASD.

Children who are diagnosed early have access to more interventions, resulting in better verbal cognition and general expression at school age and, as a consequence, they need less ongoing support at school than children diagnosed later^23^. However, it is considered that factors such as less severe symptoms, social communication difficulties at an appropriate age^24^, lack of routine surveillance and even oppressive and confusing social environments for children with ASD contributed significantly to later diagnoses^36^.

Another situation that highlights the importance of early childhood diagnosis is the underdiagnosis of cases that persist into adulthood^17^. Usually, ASD symptoms in adults manifest differently and may be more imperceptible or easily confused with other psychiatric disorders^40^. However, this lack of diagnosis can cause deficits in social integration, unemployment and mental disorders^17^, that is, they have a profound impact on quality of life.

A recent survey conducted in China listed the main factors that lead to the misdiagnosis of autistic children. The main factor listed would be the lack of knowledge about ASD on the part of health professionals, educators and the general population. Differences in knowledge on the subject were related to age, education and occupation. Younger people were the ones who knew more about the subject due to the internet and social media^36^.

On the other hand, education and profession also influenced the depth of knowledge on the subject and, as expected, health professionals were more informed than the general population. Lack of knowledge about the initial manifestations of ASD is associated with low likelihood of receiving a diagnosis. In addition, the stereotype and common sense that ASD manifests itself homogeneously in all autistic people is also a determining factor for diagnostic failure^36^.

However, there is also a discussion about what is considered early in terms of intervention for ASD. The dissemination of this information is essential to guide health professionals and avoid delays in diagnoses, since ASD symptoms and language deficits occur at school age and reach other areas of development.

In this sense, a recent study selected patients with a possible diagnosis of ASD from 12 months to four years of age and instituted therapies both for language and communication, as well as for repetitive and stereotyped behaviors, in order to then visualize which age would have the most benefit from the interventions. As a result, it was observed that 3-year-old children, when compared to 4-year-olds, had a very significant improvement in social skills. The same was observed with children over four years of age, who showed less significant progress in this area.

Accurately quantifying the presence of autistic symptoms and language ability through the Gilliam Autism Rating Scale (GARS-3) and Estimated Speech and Language Development (ESLD) tests, respectively, the non-randomized study^24^ with a sample of 40 children demonstrated a statistically significant difference in symptom reduction post-intervention between children who started therapy at three and four years of age in repetitive behavior (F=5.397, p=0.02), social interaction (F=11.19, p=0.002), and social communication (F=6.70, p=0.01), but with a non-significant difference in emotional reactions (F=3.60, p=0.06) and language ability (F=2.06, p=0.626). Patients with other neurological disorders, visual, auditory, or motor impairments were excluded from the study, and the study had a dropout rate that we considered low given the duration of the study. In the initial assessment evaluating repetitive behavior, social interaction, and social communication, there was no statistically significant difference between the groups (p>0.05) prior to early intervention.

Thus, using the fact that from 24 months of age the child begins to produce words with greater meaning and that the following six months are of great importance for the development and consolidation of language skills, in addition to adaptive behavior, the study emphasized the need for early intervention to avoid intercurrences in the child’s development potential.

In addition, other evidence demonstrates that intervention before 24 months has a potentiated effect on the ASD prognosis change. This seems to happen mainly due to intervention variations and increased heterogeneity after 24 months of age. Lombardo et al.^25^, in a study comparing the age of intervention onset - before 24 months and after 24 months of age - reported a significant age effect (F=134.09; p=2.22e-16) and a significant effect based on the group they belonged to (>24 months or <24 months of age) (F=20.92; p=5.47e-5), indicating that the age of the children at the start of treatment influenced the obtained results.

The findings are limited due to the correlational nature of the study, which employed mixed intervention methods, requiring caution in the interpretation and generalization of these results. Additionally, it lacked blinded assessors and had a small sample size of only 41 children, without providing a detailed description of the baseline characteristics of this sample. However, although limited, it brings important results to be taken into account that indicate a favorable approach before 24 months of age.

Therefore, based on the mechanisms of neuroplasticity, when stimulating the development of children with ASD early, since the brain is even more adaptable in child patients and their neural networks are able to reorganize themselves, as well as due to the characteristics under which ASD seems to develop with advancing age, EI may be able to provide greater changes in prognosis when applied before 24 months of age.

Taking into account the factors discussed above, the implementation of public policies such as training and improvement programs for education professionals and other professionals working with potential cases of ASD is likely to expand access to early screening and diagnosis, favoring a reduction in the age of intervention initiation, which we have already suggested to be beneficial before 24 months of age.

### Diagnostic and therapeutic methods of early intervention

Screening and early diagnosis protocols appear to be an important and effective tool in reducing the time between the ideal age to establish diagnosis and the actual age at which individuals receive it. In this sense, understanding the capabilities and effectiveness of each test as applied in different contexts can contribute to their effective application in the population, providing important gains in the prognosis of the disorder by reducing the main symptoms in the long term.

Thus, the Modified Checklist for Autism in Children (M-CHAT) is the most studied tool within the ASD screening level 1, used in primary health care^38^ ^,^ ^41^. This tracking tool is answered by the parents of children between 16 and 30 months of age. Considering the rate of false positives when using this method, a follow-up interview with repetition (M-CHAT-R/F) was developed, which mitigated this problem. The use of this scale proved to be quite satisfactory for tracking ASD, reducing the age at diagnosis by two years, in addition to detecting cases of underdiagnosis at 30 months of age, with a positive predictive value (PPV) of 0.72 for ASD^38^, slightly higher when compared to previous studies.

In that study, Jonsdottir et al.^38^ applied this type of screening to 1,580 children with a mean age of 32.08 months. Children at high risk of ASD were referred to a reference center for confirmation of the diagnosis through the application of the Observation Protocol for Diagnosis of Autism (ADOS-2) by specialized professionals. There was an average time of 18.28 months between the screening and definitive diagnosis. Ho ever, the study has some limitations, mainly due to the fact that some parents (n=60) did not accept to participate in the screening. This was attributed to the parents’ self-perception, as they did not consider their children to have an atypical development. Furthermore, important factors such as the characteristics of the included parents, and whether they were comparable to parents in the general population, were not taken into account, which constitutes a limitation. The study was careful in ensuring the application of the diagnostic test by expert professionals, but it failed to make important quantitative comparisons between the final screening results and the final diagnosis.

In addition to the M-CHAT-R/F, other screening tools can be applied by people without special training, such as the Ages and Stages Questionnaire (ASQ-3) and the Social Communication Questionnaire (SCQ), which are effective to detect ASD in the first years of life^39^ and, therefore, are able to provide measures of EI. The Infant Autism Observation Scale (AOSI) also seems to be significant for this review, since, through a semi-structured assessment that aims to observe behaviors typically associated with ASD risk, this scale is capable of detecting, at 14 months of age, a future diagnosis at age three^38^.

We emphasize that there are specific level 1 screening tests for the adult population, such as the Autism Spectrum Quotient (AQ), capable of effectively identifying traits of autism in this age group (PPV=0.84)^42^, successfully recognizing cases of underdiagnosis.

From this perspective, the positive screening of ASD cases makes it possible to refer them to a level 2 approach, that is, this individual is evaluated and monitored by specialized professionals. This practice saves time, costs and human resources, which may be relevant for the implementation of EI measures in developing countries.

Among the instruments used by professionals for the diagnosis of ASD, the most recognized seem to be the ADOS-2 and the Revised Diagnostic Interview for Autism (ADI-R). The ADOS-2 is an observational protocol capable of measuring, in a structured and objective way, social affection and restricted and repetitive behaviors, while the ADI-R is a structured interview carried out by professionals with the parents of children with a diagnostic hypothesis of ASD^19^ ^,^ ^26^. Both require administration by a specialist and have demonstrated robust psychometric properties for ASD diagnosis^43^.

It is evident that each case of ASD requires management with its specificities and specific individualities. This happens precisely because of the different degrees of the spectrum that an individual can find themselves in, that is, because of the heterogeneity of symptoms that each person manifests, also taking into account care for the desires and needs of the person with ASD and caregivers.

There are several successful EI programs based on scientific evidence. Currently, there are two well-established groups of EI. One is related to the theory of Applied Behavior Analysis (ABA) that uses three fundamental pillars: learning, motivation and positive reinforcement. The other is a method linked to structured education that focuses on visual work, known as Treatment and Education of Autistic (TEACCH)^44^. Both strategies integrate parents or guardians into the intervention process.

Among the treatment strategies, Early Intensive Behavioral Intervention (EIBI) had a significant prevalence in the articles selected for this review. EIBI is a method based on ABA, which analyzes specific behaviors of patients with ASD and individualizes their therapeutic goals, gradually dividing the intervention into behaviors, with positive reinforcements until the desired behavior is obtained. This method uses repeated discrete attempts to modify behavior and, normally, recognizes the patient’s successes with two distinct positive reinforcements: a primary one, for example, a food preferred by the individual, and a secondary one, which is usually a verbal compliment.

A promising longitudinal study conducted by Frazier et al.^28^ analyzed the results of the EIBI in one of the important symptoms of ASD. The language acquisition trajectory of a total of 131 children with a confirmed diagnosis of ASD was analyzed in five serial assessments covering the period from the beginning of the intervention to the end of the program. The evaluations were conducted by trained professionals and the evaluation instruments were adequate and had strong internal consistency and reliability, as reported in the current literature.

In the EIBI program, children received an average of 30 hours per week of intervention in the classroom and were divided into three groups based on whether they required educational support from an assistant or not. All groups had substantial gains in the five aspects of language evaluated (total, expressive and receptive language, receptive and expressive vocabulary), with a linear slope ranging from 1.09 to 1.37. Accompanying these estimates, the standard deviation of the estimate standard error (SE) was reported to range from 0.14 to 0.17. The p-values associated with these estimates were all less than 0.001, which indicates high statistical significance^28^.

However, we consider it important to interpret these results with caution given some important limitations, mainly because this is not a randomized study and uses a convenient clinical sample. Other important aspects are the characteristics of the study sample and the characteristics of the participants, with a high proportion of individuals with significant cognitive and developmental delay and with severity assessed as moderate or severe for ASD. Furthermore, the use of multiple measures may have limited the relationships between ASD severity and language trajectory.

The EIBI, with all the caveats, proved to be a significant EI method, with substantial gains not only in language, but also in social interaction (eye contact) and joint attention^29^ ^,^ ^31^, possible increase in Intelligence Quotient (IQ), non-verbal IQ and academic performance^30^. However, the starting age for the application of this method was also relevant, and each year of delay in the intervention using EIBI resulted in important losses in language gain when compared to other children who received the method^28^. When compared to other eclectic intervention methodologies, ABA-based therapies showed greater changes in prognosis, with the greatest benefits already observed in the first year of intervention^31^.

Beneficial results in social interaction and joint attention were demonstrated by MacDonald et al.^29^ in a study of 83 children diagnosed with ASD who received intensive intervention for 20 to 30 hours a week. The interrater reliability and agreement of the assessment process for results excellence were evaluated and demonstrated to be substantial for all measures. The assessment took place at two time points: at program entry and one year after the implementation of EIBI. Children diagnosed with ASD were compared to children with typical development (TDC) who, at program entry, outperformed them in all measures (p<0.01), except for joint attention.

To indicate and compare the results, the authors considered the standard deviation (SD) values for each age group among children with ASD and TDC. High responders were defined as those within 1 SD of their peers, average responders as those between 1 and 2 SD below their peers, and low responders as those 2 SD below the normative average.

The most significant change was observed in cognition for all age groups. Additionally, the group with an age range of 18 to 24 months exhibited the highest percentage variation in all measures compared to their TDC, with notable improvements in cognition scores (SD:7.21; t:9.93; p<0.01), followed by enhancements in early joint attention (SD:1.87; t:5.04; p<0.01) and eye contact (SD:1.14; t:4.93; p<0.01), except for stereotypy, which did not yield significant results in any of the groups. This finding regarding the age of entry in EIBI is consistent with those reported by Maksimović et al.^24^ and Lombardo et al.^25^, as previously discussed.

The study is limited by the lack of a standardized comparison sample and the use of limited or eclectic methods, which would make the results more robust for the application of EIBI. Additionally, potential confounding factors are poorly explicit, such as the socioeconomic conditions of the involved families, whether the participants had access to other interventions outside the program or not, and other factors like retention rate.

Howard et al.^31^, however, despite not conducting a randomized controlled study, overcame the aforementioned obstacles and proceeded to describe results of the intensive method in cognition (M:27.44; SD:14.18; p<0.01) and communication (M:26.23; SD:21.27; p<0.01), superior to other eclectic methods which, although also achieving success, were statistically lower for cognition (M:8.44; SD:15.04; p<0.05) and communication (M:3.57; SD:17.96; p>0.05).

In the same vein, Waters et al.^30^ conducted a non-randomized study with 94 children divided into two groups: one group received EIBI for 40 hours per week over a period of three years (n=48), while the other group received an eclectic intervention methodology. The assessments were well-structured, employing appropriate tests, and there was a high retention rate, as indicated by the low number of participants who did not complete the program (n=2). The results were significant, and the mean IQ of the EIBI group was higher in all assessments compared to the eclectic intervention group. Data analysis revealed a significantly higher monthly compared IQ score of 0.364 [95% confidence interval - CI 0.149 to 0.580], with a significant p-value of p<0.01, indicating a statistically significant relationship. In this study, baseline age was similar between groups; however, baseline IQ was slightly higher for the EIBI group (M: 64.4) compared to the eclectic intervention group (M: 58.2, t[92]=1.85), but not statistically significant (p=0.07), possibly having little impact on the study results.

Another constantly cited method is the Denver model of EI (ESDM), which also achieved significant results and the maintenance of such results over time, especially in receptive and expressive language. Estes et al.^35^ investigated, in a prospective study, the long-term maintenance of ESDM results based on the findings from a previous randomized controlled study, which observed a mean gain of 82.86 (SD=22.83) in what they referred to as intellectual ability, encompassing receptive and expressive language. A statistical analysis of variance (F=16.96; p<0.001) revealed significant differences between the groups being compared. The current study demonstrated sustained effects across all evaluated domains (intellectual capacity, adaptive behavior, autism symptoms, and challenging behaviors) but reported a significant reduction in severity for the ESDM group compared to the Eclectic group. This model is designed for children aged 12 to 60 months with ASD and has a focus on social learning.

However, Vinen et al.^26^ found results for the Denver method (ESDM) even in school-age children (72 to 108 months). In this study, 59 children were divided into two groups - ESDM (n=31) and Comparison (n=28) - and the study’s baseline was balanced, ensuring equivalence between the treatment groups regarding relevant characteristics and the initially analyzed variables of interest. Although no superiority of ESDM was found in terms of effects on severity reduction and other symptoms compared to other methods, it demonstrated that the method had an effect when compared to the baseline and was equivalent to other eclectic methods. The application of this EI tool provided significant gains in IQ, global communication, motor and social skills, the latter shown even in studies that used the evaluation of electroencephalographic (EEG) activity, proving to be an effective method of EI^45^.

Although the results obtained with the application of these methods do not allow the children submitted to them to measure and behave similarly to those of individuals without a diagnosis of ASD, both the EIBI and the ESDM, with their particularities, proved to be effective EI programs, particularly regarding language and symptoms. ESDM demonstrated a greater effect in terms of improving IQ, while EIBI proved to be more effective in improving symptoms^46^.

### Social determinants, environment and early intervention

A study carried out in the United States of America (USA) revealed that a person with ASD costs an average of US$ 1.4 million, and in cases where there is an intellectual disability that interferes with the development of skills related to the independence of that individual over his lifetime, this figure is estimated to reach US$ 2.4 million, amounting to approximately US$ 236 billion per year. Health economists believe that, by 2025, adding all expenditures, annual costs with ASD will reach US$ 461 billion^40^ ^,^ ^45^.

Still in the United Kingdom (UK), it is estimated that the cost of treatments in childhood can reach £ 31 billion per year, surpassing the amount spent on treatments for other pathologies such as asthma, diabetes and other types of intellectual disability. It is important to note that factors such as adequate access to EI improve the child’s long-term outcomes and reduce lifetime costs for the individual, family and society. This demonstrates the need to seek effective methods that, at the same time, do not require high costs and human resources^32^ ^,^ ^45^.

It is estimated that most children diagnosed with ASD are from low-income countries, mainly in South Asia, where there is the largest number of children with ASD in the world, with approximately 5 million children aged between two and nine years old in India. The fact that the largest number of children with ASD reside in developing countries makes it difficult for this population to receive the necessary and adequate treatment. Some do not even receive intervention for ASD, which is due, among other reasons, to a lack of financial and human resources^32^.

It is worth mentioning that the EI programs mentioned in the previous topic provided evidence of concrete results when applied at high intensity, that is, over 20 hours a week and in places rich in resources^33^. There is a concern about the efficiency of these programs when considering this high workload of EI application from the perspective of both cost and human resources, as these methods require qualified professionals for implementation.

In this sense, a recent study in Germany developed an EI program with a naturalistic characteristic (NDBI) lasting two hours a week, that is, of low intensity. This method, known as the Frankfurt Early Intervention Program (A-FFIP), has shown success, although further studies are still needed^34^. However, it has already been demonstrated that it is possible to perform an effective EI at low intensity.

Another alternative that seems to circumvent this problem is the application of home EI programs and intervention mediated by parents. The home modality of EI occurs after training carried out by professionals, in a home environment, through recreational activities for about two hours a day^33^. Intervention mediated by parents, in turn, seeks to train parents to understand when babies communicate^38^. These programs proved to be effective and inexpensive EI methods, with a significant improvement in the assessment of eye contact in children with ASD^33^ and communication between parents and children^38^, which can significantly impact the prognosis of individuals with ASD.

As we have already demonstrated, several screening questionnaires use parents’ experiences with children for possible diagnosis. Furthermore, it was observed that EI has a significant impact on the prognosis of ASD. However, when there is greater parental involvement in therapies, the results tend to be better, even contributing to the acceptance and understanding of the child’s diagnosis^27^ ^,^ ^37^ ^,^ ^45^. Programs that train the family to use strategies that help in child interaction contribute to the reinforcement and maintenance of acquired skills. This shows how important family participation is, so that the intervention can have better results. In most cases, it is the family who recognizes when a child starts exhibiting signs of possible developmental delays, which shows us how their report can contribute to the conclusion of a possible diagnosis.

Measures being employed to address the treatment gap in children residing in low- and middle-income countries (LMICs) include the adaptation of interventions. These interventions are tested in high-income countries to ensure their suitability for implementation by non-specialized professionals in LMICs. An EI program called Preschool Autism Communication Trial (PACT), used in the UK, which focuses on parent-mediated communication, has shown several positive effects on social outcomes and improved communication. This is a method that can be viable and effective in situations involving sociocultural differences, adapting to local beliefs, and can be used by non-specialized professionals.

Rahman et al.^32^, in an effort to apply the effects already obtained and documented by the Parent-Mediated Autism Communication Therapy (PACT) method, this time in low-income countries, conducted a single-blind randomized study comparing the outcomes of 12 sessions of the PACT with usual treatment, in 65 children aged two to nine years from India and Pakistan in the South Asia region, randomly selected through the use of probabilistic minimization. The baseline of the study demonstrated a balance between the intervention groups, with comparable relevant characteristics and variables. The Parent-Mediated Autism Communication Therapy for South Asia (PASS) utilizes a similar approach to the previously mentioned PACT, but is adapted to the local culture and language where it is being implemented. The results of this study revealed an improvement in communication between the children and their parents, reinforcing that parent-mediated intervention can indeed be effective in low- and middle-income countries. It is important to highlight that family adherence and involvement are crucial for these results to occur.

Compared to PACT, PASS demonstrated greater effectiveness both for parent-child synchrony (effect size 1.61 [95% CI 0.90 to 2.32]) and child communication (effect size 1.61 [95% CI 0.90 to 2.32]), which are attributed to the higher level of parental support present in PASS. However, regarding shared attention, the treatment outcome was negative compared to PACT (effect size -0.70 [95%CI -1.16 to -0.23]). The study presented cohesive and important results for the implementation of this type of method in LMICs.

Therefore, it is evident that the study of the application of intervention programs in developing countries can have a significant effect on the implementation of EI in these regions and, for all the reasons already explained, positively affect the prognosis of people with ASD^32^. In addition, the implementation of measures of parental involvement in the diagnosis and treatment of children was recognized as a beneficial factor for both the patient and his/her guardians, making this practice essential for reducing the time of diagnosis and application of EI, which promotes an improvement in the prognosis of patients with ASD.

The implementation of EIBI or ESDM is therefore favorable for prognostic gains when analyzing certain symptoms of ASD, as discussed. We also suggest that the implementation of programs similar to PACT or PASS in regions with low socioeconomic status can overcome the economic barrier through sustainable utilization of human and financial resources, while also achieving prognostic gains that would be unsustainable in other situations or eclectic methodologies in those localities.

The synthesis of the main persistent challenges in the implementation of EI, as well as the effects of this persistence on the prognosis of patients with ASD, with a social approach, is unprecedentedly described in this review. We believe that this could contribute to the implementation of public policies in the field of early intervention and the conduct of new studies that address persistent gaps, such as a more comprehensive application of intervention methods for LMICs, quantification of interference in the severity of ASD, and novel approaches involving parents more actively in the treatment of ASD.

Furthermore, the discussions generated here strongly suggest the importance of different professionals in implementing intervention measures. Most of the studies presented involved the participation of other health agents, such as psychologists and nurses, on the front line of identifying warning signs and interventions for ASD. Education professionals are those who have the most contact with the population at risk of this disorder and, in addition, were present throughout the implementation of the proposed interventions in the school environment. This demonstrates the importance of the multidisciplinary team in the care of individuals with ASD and, also, the alignment between these professionals in a coordinated therapeutic plan.

In conclusion, EI is able to significantly influence the prognosis of patients with ASD. The most important changes involve the improvement of cognition, the acquisition of social skills and the reduction of stereotyped behaviors, and the ideal age for diagnosis and initiation of intervention was before 24 months of age. The benefits of EI involve changes in prognosis and may even influence social issues, since the results showed less need for school monitoring for these children, and a gain in functionality and social integration.

There are still challenges in implementing EI programs, including:

- knowledge of the main screening, diagnosis and intervention tools for ASD;
- the still high costs of implementing EI strategies;
- the effective involvement of parents in the stages of EI programs; and
- recognition of signs of ASD before 24 months of age.

The implementation of measures that impact these axes, especially in places lacking specialized professionals, can be efficient measures to reduce the impacts of this disorder on populations in developing countries.

**Figure 3.**
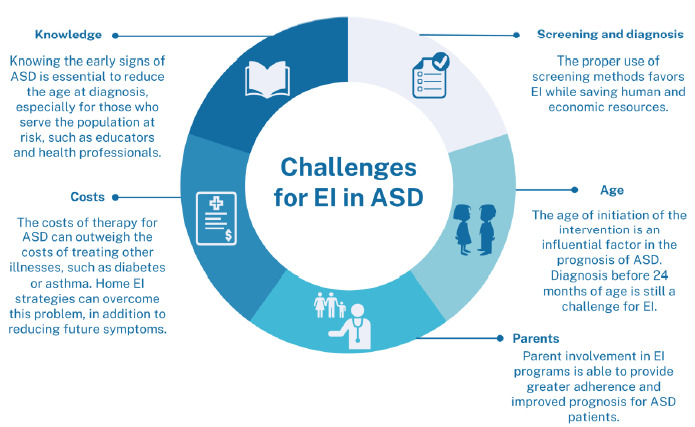
Challenges for early intervention in autism spectrum disorder.

This review is unprecedented and innovative in that it summarizes and categorizes the main challenges encountered in implementing EI techniques for autism (Figure 3). At the same time, it establishes links between these challenges and the potential impact on various aspects of prognosis. We recognize that these connections are crucial to the practicality of this study and any other, serving as guidance for the development of public policies and social actions.

Thus, despite clarifying significant results, this review presents limitations that deserve consideration. In this regard, the predefined search period for this review restricted its results, leading to a relatively small number of randomized clinical trials included. This, in turn, reduces its level of evidence, without, however, invalidating it.

Furthermore, the selected studies limited our review, as they have a small sample size and lack application in heterogeneous populations and those with low socioeconomic status. Therefore, quantifying the impacts of each of these axes of challenges we listed still requires further evidence and investigation.

Despite this, a significant strength of this review is the inclusion of populations with low socioeconomic status, despite their limitations. This inclusion is important because it not only highlights the scarcity of clinical studies in this area but, more importantly, it contributes to scientific discourse by shedding light on the specific, but analogous, challenges faced by these vulnerable populations.
